# Effect of Sex on the Physicochemical, Technological, and Nutritional Quality of Longissimus Dorsi Muscle in DanBred Hybrid Pigs

**DOI:** 10.3390/ani16203281

**Published:** 2026-10-18

**Authors:** Karolina Dobiesz, Wojciech Kozera, Grzegorz Żak, Magdalena Szyndler-Nędza, Aurelia Mucha, Pavol Findura, Miroslav Pristavka, Renata Lewko-Wojtowicz, Jarosław Krzysztoń, Krzysztof Karpiesiuk

**Affiliations:** 1 Department of Pig Breeding, Faculty of Animal Bioengineering, University of Warmia and Mazury in Olsztyn, ul. Oczapowskiego 5, 10-719 Olsztyn, Poland; karolina.dobiesz@agriplus.pl (K.D.); kozwoj@uwm.edu.pl (W.K.); 2 Department of Pig Breeding, National Research Institute of Animal Production, 32-083 Balice, Poland; grzegorz.zak@iz.edu.pl (G.Ż.); magdalena.szyndler@iz.edu.pl (M.S.-N.); aurelia.mucha@iz.edu.pl (A.M.); 3 Department of Machines and Production Biosystems, Faculty of Engineering, Slovak University of Agriculture in Nitra, Tr. A. Hlinku 2, 949 76 Nitra, Slovakia; pavol.findura@uniag.sk; 4 Faculty of Agriculture and Technology, University of South Bohemia in České Budějovice, 370 05 České Budějovice, Czech Republic; 5 Department of Quality and Engineering Technology, Faculty of Engineering, Slovak University of Agriculture in Nitra, 949 76 Nitra, Slovakia; miroslav.pristavka@uniag.sk; 6 Ceva Animal Health Polska, ul. Okrzei 1A, 03-715 Warszawa, Poland; renata.lewko@ceva.com; 7 Agri Vet Sp. z o.o., ul. Marcelińska 92, 60-324 Poznań, Poland; jaroslaw.krzyszton@agriplus.pl

**Keywords:** pork quality, longissimus dorsi, sex, gilts, barrows, entire males, fatty acid profile, vitamins, cholesterol, meat texture

## Abstract

The aim was to evaluate the effect of sex on the quality of meat obtained from DanBred hybrid pigs. Samples of the longissimus dorsi (LD) muscle were collected from three groups: gilts, barrows and entire males (10 animals per group). Animals were housed under identical management conditions and fed a four-phase feeding program ad libitum. Following commercial slaughter, LD muscle samples were collected from the right carcass side. The quality of meat was evaluated by measuring pH, colour, proximate composition, fatty acid profile, vitamins A and E, cholesterol content, sensory characteristics, and texture parameters. Significant differences among groups were observed for loin eye depth. Meat from gilts and barrows demonstrated superior technological quality. Dry matter content was significantly higher in barrows than in entire males and was also higher in gilts than in entire males. Entire males exhibited the lowest intramuscular fat content. No significant differences were found in ultimate pH among the groups. The highest proportion of unsaturated fatty acids was observed in barrows, whereas significant differences in vitamin and cholesterol contents were associated with sex. These findings demonstrate that sex significantly affects several technological and nutritional characteristics of pork. Further studies involving larger experimental populations are warranted to confirm the results.

## Simple Summary

Improving production efficiency and pork quality is a major objective of pig production. One solution is gender-segregated breeding. The objective of this study was to compare the physicochemical, technological, sensory, and nutritional quality of the longissimus dorsi muscle obtained from gilts, barrows and entire males of DanBred hybrid pigs. Ten muscle samples were collected from right carcasses from each of the analyzed groups. The present study demonstrated that sex significantly influenced selected carcass characteristics, meat quality traits, fatty acid composition, vitamin content, and texture properties. The results indicate that gilts and barrows produced pork with more desirable technological, sensory, and nutritional characteristics than entire males under the production conditions applied in this study. These findings may contribute to the optimization of pork production systems and provide useful information for producers and the meat industry when selecting production strategies for DanBred hybrid pigs.

## 1. Introduction

Modern pig production aims not only to maximize growth performance and production efficiency but also to improve the quality of pork destined for consumers and the meat industry [1,2]. Among the numerous factors (e.g., genotype, nutrition, stressors, and environmental conditions) affecting pork quality, the sex of the animal plays a particularly important role because it influences growth rate, carcass composition, fat deposition, and muscle metabolism [3,4]. Consequently, sex-related physiological differences may substantially affect both the technological and nutritional quality of pork.

The longissimus dorsi (LD) muscle is one of the most economically important muscles in the pig carcass and is commonly used to evaluate meat quality. Its physicochemical characteristics, including tenderness, colour, water-holding capacity, and intramuscular fat content, are major determinants of consumer acceptance and processing suitability.

Commercial pig production generally involves three sex categories: gilts, surgically castrated males (barrows), and entire (uncastrated) males. Each group differs in endocrine status and metabolic activity, resulting in differences in carcass composition and meat quality. Gilts generally exhibit favourable carcass characteristics and moderate intramuscular fat deposition, which contribute to desirable sensory properties. Entire males are characterized by improved feed efficiency, leaner carcasses, and faster growth; however, their meat is more susceptible to boar taint and often exhibits lower fat content, potentially reducing eating quality. Although surgical castration effectively eliminates boar taint, it promotes greater fat deposition and may influence carcass composition and meat quality depending on the production system and intended end use [5].

Previous studies have demonstrated that sex-related physiological differences affect several quality traits, including muscle pH, colour, water-holding capacity, marbling, texture, and fatty acid composition [6,7]. These characteristics are largely determined by post-mortem biochemical processes, particularly glycogen metabolism and protein denaturation, which directly influence technological and sensory properties of pork [8].

Increasing societal concerns regarding animal welfare and the progressive reduction in the use of surgical castration have intensified interest in alternative production systems and their impact on meat quality [3,9]. Therefore, comprehensive comparisons of meat quality among gilts, barrows, and entire males are essential for developing production strategies that balance animal welfare, production efficiency, and consumer expectations.

The objective of this study was to compare the physicochemical, technological, sensory, and nutritional quality of the *Musculus longissimus dorsi* obtained from gilts, surgically castrated males, and entire males of the DanBred hybrid. Emphasis was placed on meat composition, fatty acid profile, vitamin and cholesterol contents, texture characteristics, and sensory attributes to determine the influence of sex on pork quality.

## 2. Materials and Methods

### 2.1. Animals and Experimental Design

The study was conducted using 30 DanBred hybrid pigs divided into three experimental groups according to sex: gilts, surgically castrated males (barrows; castrated within the first 7 days of life), and entire males (10 animals per group). Samples of the longissimus dorsi (LD) muscle, approximately 10 cm in thickness, were collected from each animal after slaughter. Ethical approval was not required because the study was conducted exclusively on tissues collected from animals slaughtered under commercial conditions. No experimental procedures were performed on live animals beyond routine production practices. The experiment complied with Directive 2010/63/EU of the European Parliament and of the Council on the protection of animals used for scientific purposes. Animal housing and management complied with current European Union and Polish legislation governing pig welfare. Permission to collect biological material and use production data was obtained from the farm owners. The pigs were slaughtered in commercial abattoirs following standard industry procedures and in accordance with Council Regulation (EC) No. 1099/2009 on the protection of animals at the time of killing and the relevant national legislation.

### 2.2. Animal Management and Feeding

Animals were selected before the start of the fattening period according to sex and housed in separate buildings. Fattening began at an average body weight of approximately 32 kg and was completed when pigs reached a final body weight of 126 ± 5 kg. The average fattening period lasted 94 days. Animals were housed under commercial conditions in fully slated pens accommodating 50 pigs per pen. Feed and water were available ad libitum throughout the experiment. A four-phase feeding program based on complete diets formulated according to the recommendations of the Seges Pig Research Centre (SPRC) was applied:

• Finisher 1: 23–43 kg body weight;
• Finisher 2: 43–63 kg body weight;
• Finisher 3: 63–85 kg body weight;
• Finisher 4: above 85 kg body weight until slaughter.

The ingredient composition and chemical composition of the experimental diets are presented in Table 1 and Table 2. The chemical composition and nutritional values of the complete diets presented in Table 1 and Table 2 were obtained from the manufacturer’s declarations and were not determined by laboratory analyses in the present study.

### 2.3. Slaughter Procedures and Carcass Evaluation

Commercial slaughter was performed in accordance with current animal welfare legislation. Immediately after slaughter, lean meat percentage, backfat thickness, and loin eye depth were determined on the warm right carcass side using a SYDEL CGM ultrasonic carcass grading system (SYDEL, Lorient, France). Measurements were taken between the third and fourth ribs from the last rib at a distance of 6 cm from the dorsal midline according to the manufacturer’s recommendations.

### 2.4. Muscle Sampling

Following 24 h of carcass chilling at 4 °C, LD muscle samples were collected from the right carcass side between the third and fourth lumbar vertebrae. Animals selected for laboratory analyses represented each experimental group and had comparable slaughter body weights. All samples were collected on the same slaughter day to minimize environmental variation.

### 2.5. Determination of pH and Meat Colour

Ultimate pH (pH24) was measured 24 h postmortem using a WTW 340 pH meter (WTW Wissenschaftlich-Technische Werkstätten GmbH, Weilheim, Germany) equipped with a Hamilton Double Pore glass electrode. The instrument was calibrated using standard buffer solutions of pH 4.01 and 7.00 before and during measurements. Meat colour was determined on freshly cut LD muscle surfaces using a MiniScan XE Plus spectrophotometer (HunterLab, Reston, VA, USA) equipped with a D65 illuminant and a 10° standard observer. Colour parameters were recorded in the CIE L a b* colour space over the wavelength range of 400–700 nm after calibration against standard white and black reference tiles.

### 2.6. Proximate Chemical Composition

The proximate composition of LD muscle was determined according to AOAC [10] procedures. Analyses included dry matter, crude protein, intramuscular fat, and ash content. The analyses were performed at the laboratory of the Department of Commodity Science and Processing of Animal Raw Materials, Faculty of Animal Bioengineering, University of Warmia and Mazury in Olsztyn, Olsztyn, Poland.

### 2.7. Fatty Acid Analysis

Fatty acid composition was determined by gas chromatography (GC-FID) using a CP-3800 gas chromatograph (Varian, Walnut Creek, CA, USA). Fatty acid methyl esters (FAMEs) were prepared according to a modified Peisker method using methanol, chloroform, and concentrated sulphuric acid (100:100:1, *v*/*v*/*v*). Chromatographic separation was performed on a capillary column (50 m × 0.25 mm × 0.25 μm). Helium was used as the carrier gas at a constant flow rate of 1.2 mL/min. Injector and detector temperatures were maintained at 225 °C and 250 °C, respectively, whereas the column temperature was 200 °C. Fatty acids were identified by comparing retention times with certified FAME standards (Sigma-Aldrich, St. Louis, MO, USA). Results were expressed as percentages of total detected fatty acids. The nutritional quality of the fatty acid profile was additionally assessed using the desirable fatty acid (DFA) and hypercholesterolemic fatty acid (OFA) indices. DFA was calculated as DFA = UFA + C18:0, where UFA represents the sum of MUFA and PUFA, whereas OFA was calculated as OFA = C14:0 + C16:0. The DFA index includes unsaturated fatty acids and stearic acid (C18:0), the latter being considered relatively neutral with respect to serum cholesterol, whereas OFA comprises myristic (C14:0) and palmitic (C16:0) acids, which are associated with hypercholesterolemic effects. Consequently, a higher DFA value and a lower OFA value indicate a more favorable nutritional fatty acid profile.

### 2.8. Determination of Vitamins A and E

Retinol and tocopherol concentrations were determined by high-performance liquid chromatography (HPLC). Muscle samples were homogenized using an Ultra-Turrax T25 homogenizer (IKA-Werke GmbH & Co. KG, Staufen im Breisgau, Germany). After alkaline saponification with potassium hydroxide and ascorbic acid, vitamins were extracted using n-hexane. The extracts were evaporated under nitrogen, reconstituted in n-hexane, and analysed using a Shimadzu HPLC system (Kyoto, Japan) equipped with a reversed-phase C18 column. Retinol was quantified using UV detection at 336 nm, whereas tocopherols were detected fluorometrically (excitation 295 nm; emission 330 nm). Quantification was performed using certified external standards according to PN-EN-ISO 6867 [11] and PN-EN-ISO 14565 [12].

### 2.9. Cholesterol Determination

Cholesterol concentration was determined by HPLC following alkaline hydrolysis and solvent extraction. Homogenized muscle samples were saponified with potassium hydroxide in ethanol containing L-ascorbic acid. Cholesterol was extracted using a mixture of n-hexane and ethyl acetate, evaporated to dryness, dissolved in the mobile phase, and analysed using a Shimadzu HPLC system equipped with a C18 column and UV detector operating at 222 nm. Quantification was based on an external cholesterol standard (Merck, Darmstadt, Germany).

### 2.10. Sensory Evaluation

Sensory evaluation was performed using cooked LD muscle samples free of external fat and connective tissue. Samples weighing approximately 50 g were heated in 0.62% sodium chloride solution until an internal temperature of 75 °C was reached. Samples were coded with random three-digit numbers and presented to a trained five-member sensory panel under controlled laboratory conditions at room temperature. The following attributes were evaluated using a five-point scale:

• Odour intensity;
• Odour acceptability;
• Tenderness;
• Juiciness;
• Flavour intensity;
• Flavour acceptability.

### 2.11. Texture Analysis

Texture measurements were performed using a TA.XTplus Texture Analyser (Stable Micro Systems, Godalming, UK). Frozen samples were thawed, cooked to an internal temperature of 80 °C, and cooled for 24 h at 4 °C, and cylindrical cores were prepared parallel to the muscle fibres. Warner–Bratzler shear force (WBSF) was determined using a triangular blade attachment at a crosshead speed of 4.5 mm/s. Maximum shear force (N) and shear energy (N/s) were recorded. Texture Profile Analysis (TPA) was conducted by double compression to 70% of sample height using a cylindrical probe. The following parameters were calculated: hardness, springiness, cohesiveness, resilience, and chewiness [13].

### 2.12. Statistical Analysis

The experimental data were analysed using the following linear model:

Y_ij_ = µ + a_i_ + cov_(x)_ + e_ij_

where y_ij_—observation ij, µ—mean value, a_i_—fixed effect of ith sex (1, 2, 3), cov_(x)_—concomitant variable for slaughter body weight (x), and e_ij_—random error.

Normality of residuals was verified using the Kolmogorov–Smirnov test, whereas homogeneity of variance was assessed using Levene’s test. Differences among treatment means were evaluated using Fisher’s least significant difference (LSD) test. Statistical significance was declared at *p* ≤ 0.05. All statistical analyses were performed using STATISTICA version 14.1 (StatSoft Inc., Tulsa, OK, USA).

## 3. Results

### 3.1. Slaughter Performance and Carcass Characteristics

The effects of sex on slaughter performance and carcass characteristics of DanBred hybrid pigs are presented in Table 3. No significant differences were observed among the experimental groups in final body weight, lean meat percentage, or backfat thickness. However, carcass weight and loin eye area were significantly affected by sex. Both gilts and barrows produced significantly heavier carcasses than entire males (*p* < 0.05), with no significant difference in carcass weight between gilts and barrows. Likewise, gilts exhibited the largest loin eye area, which was significantly greater than that of entire males (*p* < 0.01). Barrows also had a significantly larger loin eye area than entire males (*p* < 0.05). These results indicate superior muscularity in gilts and barrows despite the absence of significant differences in final body weight; both groups also showed numerically greater backfat thickness than entire males, although these differences were not statistically significant. Overall, the present results indicate that sex had only a limited effect on the slaughter performance of DanBred hybrid pigs. Although carcass weight and loin eye area differed significantly among sex groups, final body weight, lean meat percentage, and backfat thickness remained comparable under the experimental conditions applied in this study.

### 3.2. Physicochemical Characteristics and Proximate Composition of the Longissimus Dorsi Muscle

The physicochemical characteristics and proximate composition of the longissimus dorsi muscle are presented in Table 4. Sex significantly affected several quality traits, particularly meat colour and chemical composition, whereas ultimate pH, ash content, and intramuscular fat content did not differ significantly among the experimental groups. The highest dry matter content was recorded in barrows, followed by gilts, whereas the lowest value was observed in entire males. Dry matter content was significantly higher in barrows than in entire males (*p* < 0.01) and was also higher in gilts than in entire males (*p* < 0.05). Similarly, the longissimus dorsi muscle of barrows contained significantly more crude protein than that of entire males (*p* < 0.05). In contrast, ash and intramuscular fat contents were not significantly affected by sex, although entire males tended to exhibit the lowest intramuscular fat concentration. Ultimate pH (pH24) ranged from 5.26 to 5.30 and did not differ significantly among the experimental groups. These values indicate normal post-mortem glycolysis and exclude the occurrence of PSE (pale, soft, exudative) pork, as the ultimate pH remained within the range considered typical for high-quality pork. Significant differences were also observed in meat colour. Barrows exhibited the highest lightness (L*) values, whereas meat from entire males was significantly darker (*p* < 0.05). No significant differences were found in redness (a*), although the lowest values were recorded in entire males. In contrast, yellowness (b*) was significantly lower in entire males than in gilts and barrows (*p* < 0.01). These findings indicate that pork from entire males was darker and less yellow than that obtained from gilts and barrows.

### 3.3. Fatty Acid Profile of the Longissimus Dorsi Muscle

The fatty acid composition of intramuscular fat in the longissimus dorsi muscle of DanBred hybrid pigs is presented in Table 5. Sex significantly affected the concentrations of several individual fatty acids as well as the overall fatty acid profile. Among the saturated fatty acids, entire males exhibited significantly higher concentrations of pentadecanoic acid (C15:0) than gilts and barrows (*p* < 0.05). Similarly, the concentrations of margaric acid (C17:0) and stearic acid (C18:0) were significantly higher in entire males than in barrows (*p* < 0.01). In contrast, gilts had a significantly higher concentration of palmitoleic acid (C16:1 n-7) than entire males (*p* < 0.05). Entire males also exhibited significantly higher concentrations of heptadecenoic acid (C17:1) than both gilts (*p* < 0.05) and barrows (*p* < 0.01). The concentration of oleic acid (C18:1 n-9), the predominant monounsaturated fatty acid, was highest in barrows and lowest in entire males. Highly significant differences were observed between barrows and entire males (*p* < 0.01), whereas gilts showed intermediate values that differed significantly from both remaining groups (*p* < 0.05). Entire males also had significantly higher concentrations of linoleic acid (C18:2 n-6) and α-linolenic acid (C18:3 n-3) than gilts and barrows. No significant differences were found in the concentrations of gondoic acid (C20:1 n-9), eicosadienoic acid (C20:2), or arachidonic acid (C20:4 n-6). Sex also significantly affected the overall fatty acid profile. Barrows were characterised by the highest proportion of unsaturated fatty acids (UFAs) and monounsaturated fatty acids (MUFAs), whereas entire males contained the highest proportion of polyunsaturated fatty acids (PUFAs), including n-6 PUFAs. Significant differences in n-3 PUFAs were also observed, with entire males exhibiting higher concentrations than barrows (*p* < 0.01) and gilts (*p* < 0.05). The proportion of desirable fatty acids (DFAs) was highest in barrows and lowest in gilts (*p* < 0.05). Conversely, the highest proportion of hypercholesterolemic fatty acids (OFAs) was observed in gilts, whereas barrows exhibited the lowest values (*p* < 0.05), indicating a more favourable nutritional lipid profile in surgically castrated males.

### 3.4. Vitamin A, Vitamin E Homologues, and Cholesterol Content

The concentrations of vitamin A, vitamin E homologues, and cholesterol in the longissimus dorsi muscle are presented in Table 6. Sex significantly affected the concentrations of several fat-soluble vitamins and cholesterol. Gilts exhibited significantly higher concentrations of α-tocopherol than barrows (*p* < 0.05) and significantly higher β-tocopherol concentrations than entire males (*p* < 0.05). Likewise, γ-tocopherol concentrations were significantly higher in gilts than in both barrows and entire males (*p* < 0.05). No significant differences were observed in δ-tocopherol concentration among the experimental groups. Consequently, the highest total tocopherol concentration was recorded in gilts and was significantly higher than that observed in barrows (*p* < 0.05). Retinol concentration was significantly higher in entire males than in barrows (*p* < 0.05), whereas gilts showed intermediate values. In contrast, the longissimus dorsi muscle of entire males contained significantly higher cholesterol concentrations than those of gilts and barrows (*p* < 0.01).

### 3.5. Sensory Characteristics of the Longissimus Dorsi Muscle

The sensory characteristics of the longissimus dorsi muscle are presented in Table 7. Sex significantly influenced several sensory attributes, particularly odor acceptability, flavor acceptability, flavor intensity, and juiciness. The lowest scores for odor acceptability and flavor acceptability were recorded in entire males. These differences were statistically significant compared with gilts (*p* < 0.05) and barrows (*p* < 0.01), respectively. In addition, entire males received the highest scores for flavor intensity, which differed significantly from those of gilts (*p* < 0.01). These findings suggest the presence of characteristic sensory attributes in pork obtained from entire males. No significant differences were observed in tenderness among the experimental groups. In contrast, meat from barrows was significantly less juicy than meat from gilts and entire males (*p* < 0.05).

### 3.6. Warner–Bratzler Shear Force and Texture Profile Analysis

The Warner–Bratzler shear force (WBSF) and texture profile analysis (TPA) results for the longissimus dorsi muscle are presented in Table 8. Sex significantly affected shear force, shear energy, springiness, cohesiveness, and resilience, whereas no significant differences were observed for hardness or chewiness. The meat of entire males was characterized by the highest Warner–Bratzler shear force and shear energy. Both parameters were significantly higher than those recorded for gilts and barrows (*p* < 0.01), indicating lower tenderness of pork obtained from entire males. In contrast, the longissimus dorsi muscle of gilts exhibited significantly greater springiness and cohesiveness than that of the other sex groups (*p* < 0.05). Gilts also showed significantly higher resilience than barrows (*p* < 0.05), whereas no significant differences were observed between gilts and entire males. Hardness and chewiness did not differ significantly among the experimental groups.

## 4. Discussion

### 4.1. Slaughter Performance and Carcass Characteristics

Recent studies investigating the effects of sex on slaughter performance have primarily focused on comparisons between gilts and barrows. Xia et al. [14] reported higher carcass weight, backfat thickness, loin eye area, and loin depth in gilts than in barrows. Similarly, Kozera et al. [15] demonstrated that gilts had a significantly higher lean meat percentage and lower average backfat thickness than surgically castrated males. Xie et al. [16] also reported significantly greater backfat thickness and larger longissimus dorsi muscle area in gilts than in barrows. In contrast, Alves et al. [17] and Malgwi et al. [18] found no significant differences in backfat thickness or loin eye area between gilts and barrows. These discrepancies among studies may be attributed to differences in genotype, breeding line, slaughter weight, feeding strategy, and production system, all of which are known to influence carcass composition.

### 4.2. Physicochemical Characteristics and Proximate Composition of the Longissimus Dorsi Muscle

The lower dry matter content observed in entire males is consistent with the findings of Dobiesz et al. [3], who likewise reported the lowest dry matter concentration in the longissimus dorsi muscle of entire males. Similar to the present study, these authors found no significant effect of sex on intramuscular fat content. In contrast, they reported different relationships with respect to crude protein concentration. Kozera et al. [9] observed a higher protein content in the longissimus dorsi muscle of barrows than in gilts; however, the differences were not statistically significant. Comparable results were reported by Bahelka et al. [19], who likewise found only minor differences in muscle protein concentration between sex groups. With regard to intramuscular fat, Kozera et al. [9] reported a higher lipid content in barrows than in gilts. Similarly, Jaturasitha et al. [20], Cai et al. [21], and Batorek et al. [22] demonstrated lower intramuscular fat levels in entire males than in surgically castrated males, confirming the marked effect of castration on lipid deposition. In contrast, Bahelka et al. [19] reported higher lipid content in the muscle tissue of entire males than in barrows. Furthermore, Aluwé et al. [23] demonstrated that surgically castrated males developed thicker backfat than gilts. Increased intramuscular fat content is generally considered beneficial because it improves the sensory characteristics and technological quality of pork [24]. The ultimate pH values obtained in the present study are comparable with those reported by Kozera et al. [9], who also observed slightly higher pH values in gilts than in barrows. However, the pH values recorded in the present experiment were higher than those reported by Dobiesz et al. [7]. Nevertheless, all values remained within the range characteristic of normal-quality pork and confirmed the absence of PSE meat. Meat colour is one of the most important quality attributes affecting consumer acceptance and purchasing decisions. It is influenced by numerous factors, including genotype, muscle characteristics, post-mortem metabolism, and production conditions [25]. Similar to the findings of Dobiesz et al. [7], meat from entire males was darker than that from gilts and barrows. In contrast, Kozera et al. [9] reported the darkest longissimus dorsi muscle in barrows, indicating that genotype and production system may substantially influence colour development. According to Trevisan and Brum [26], pork of desirable quality should exhibit a characteristic reddish-pink colour that is highly preferred by consumers. Meat colour also affects the commercial value of pork because consumers frequently associate colour with freshness and overall quality. Moreover, colour stability during retail display is influenced by biochemical and microbiological changes occurring during storage, which may ultimately affect product shelf life.

### 4.3. Fatty Acid Profile of the Longissimus Dorsi Muscle

The fatty acid composition of pork is one of the principal determinants of its nutritional value, oxidative stability, and technological suitability [27]. In addition to genotype, the fatty acid profile is influenced by numerous factors, including diet, slaughter age, sex, and the anatomical location of the analysed muscle [28]. In contrast to the present findings, Czech et al. [29] reported no significant effect of sex on the fatty acid composition of porcine muscle. Bahelka et al. [19] observed significantly higher stearic acid concentrations in barrows than in entire males, whereas Grela et al. [30] reported higher stearic acid concentrations in barrows than in both gilts and entire males. Furthermore, Bahelka et al. [19] found higher concentrations of myristic and palmitic acids in barrows than in entire males, which differs from the results obtained in the present study. The overall fatty acid profile observed in the present study also differs from that reported by Dobiesz et al. [3], who found higher proportions of saturated fatty acids (SFAs) in barrows than in gilts. Similar observations were reported by Pauly et al. [31], Mackay et al. [32], and Poklukar et al. [33]. Conversely, Kozera et al. [9] demonstrated lower SFA and higher UFA proportions in gilts than in barrows, whereas Dobiesz et al. [7] reported no significant effect of sex on the proportions of saturated and unsaturated fatty acids. Monounsaturated fatty acids are considered beneficial because their increased proportion improves the nutritional value and eating quality of pork [34]. In contrast to the present results, Kozera et al. [9] and Dobiesz et al. [7] reported no significant differences in DFA and OFA indices among sex groups. It should also be emphasised that, apart from genotype and sex, the fatty acid composition of pork is strongly influenced by nutrition, particularly by the type and source of dietary lipids included in pig diets [35,36].

### 4.4. Vitamin A, Vitamin E Homologues, and Cholesterol Content

Dobiesz et al. [37] reported no significant effect of sex on α-tocopherol concentration in the longissimus dorsi muscle, which is consistent with the statistical interpretation of their study but differs from the present findings. Furthermore, these authors reported higher β-tocopherol concentrations in entire males than in gilts, whereas muscle samples obtained from gilts contained the lowest vitamin E concentration, which is opposite to the results obtained in the present study. According to Świąder et al. [38], meat containing higher concentrations of tocopherols is characterised by superior quality and extended storage stability because tocopherols effectively retard lipid oxidation. Therefore, the higher total tocopherol concentration observed in gilts may contribute to improved oxidative stability and better storage quality of pork. The present results also differ from those reported by Dobiesz et al. [37], who found the highest retinol concentration in the longissimus dorsi muscle of barrows. Cholesterol concentrations determined in all experimental groups were lower than those reported for the Puławska breed and wild boar by Kasprzyk [39]. Likewise, the cholesterol concentrations recorded in the present study were lower than those reported by Blicharski [28]. In contrast to the present findings, Bahelka et al. [19] demonstrated significantly lower cholesterol concentrations in the longissimus dorsi muscle of entire males than in barrows. Casapu et al. [40] also reported sex-related differences in muscle lipid composition, showing that, irrespective of breed, the longissimus dorsi muscle of Landrace and Duroc gilts contained higher cholesterol concentrations than that of males. Overall, the present results demonstrate that sex significantly influences the concentrations of vitamin E homologues, retinol, and cholesterol in the longissimus dorsi muscle of DanBred hybrid pigs. The higher tocopherol concentrations observed in gilts may improve the oxidative stability of pork, whereas the elevated cholesterol concentration found in entire males represents a less favourable nutritional characteristic.

### 4.5. Sensory Characteristics of the Longissimus Dorsi Muscle

Kozera et al. [9] also evaluated the sensory characteristics of pork obtained from different sex groups but did not observe significant differences in odor intensity, odor acceptability, flavor intensity, or flavor acceptability. However, similarly to the present study, meat from gilts was characterised by greater juiciness and tenderness than that obtained from barrows. Overall, the present results indicate that sex significantly affects selected sensory characteristics of pork, particularly those related to odor and flavor acceptability, whereas tenderness appears to be less dependent on sex under the experimental conditions applied in this study.

### 4.6. Warner–Bratzler Shear Force and Texture Profile Analysis

Numerous studies have evaluated meat texture by comparing different pig breeds because texture is an important determinant of technological suitability and consumer acceptance. Florowski et al. [41], comparing the processing suitability of meat from five pig breeds, demonstrated that Duroc and Line 990 pigs exhibited the lowest shear force and compression force values, whereas Pietrain pigs were characterised by the highest values. Similar results were reported by Migdał et al. [42], who evaluated Warner–Bratzler shear force and texture profile analysis in five pig breeds and likewise found less favourable texture characteristics in Pietrain pigs. These findings indicate the lower culinary and technological suitability of meat from this breed. Similarly, Cebulska [43], who compared meat quality in two Polish native breeds and high-performance commercial hybrids, reported that Puławska pigs produced the most tender meat, as confirmed by both instrumental shear force measurements and sensory evaluation. According to Kołczak [44], tenderness is most commonly evaluated instrumentally by measuring the force required to shear a meat sample perpendicular to the orientation of the muscle fibres. Higher shear force values indicate lower meat tenderness. This relationship is consistent with the results obtained in the present study, in which meat from entire males required the greatest shear force and simultaneously received the lowest tenderness scores during sensory evaluation. Migdał et al. [42] demonstrated that shear force, hardness, and chewiness increase with animal age and reported strong correlations among these texture parameters. Such relationships may partly explain the variation in meat texture observed among different experimental groups. Relatively few studies have investigated the effect of sex on pork texture. Li et al. [45] reported no significant effect of sex or slaughter weight on the texture characteristics of pork from gilts and barrows. Similarly, Chaosap et al. [46] found no significant differences in Warner–Bratzler shear force between these sex groups. In contrast, Jeremiah et al. [47] demonstrated significant differences in meat tenderness among gilts, barrows, and entire males. In their study, pork from barrows exhibited the lowest shear force, whereas gilts showed the highest values.

The results obtained in the present study are therefore only partly consistent with those reported in the literature. These discrepancies may be attributed to differences in genotype, breeding lines, production systems, slaughter weight, and environmental conditions, all of which are known to influence meat texture and tenderness.

## 5. Conclusions

The present study demonstrated that sex significantly influenced selected carcass characteristics, meat quality traits, fatty acid composition, vitamin content, and texture properties of the longissimus dorsi muscle in DanBred hybrid pigs. Gilts exhibited greater loin eye depth than the other sex groups, whereas barrows and gilts produced carcasses with higher carcass weight than entire males. From a technological perspective, meat from gilts and barrows showed more favourable quality characteristics than meat from entire males, including lower Warner–Bratzler shear force and higher sensory acceptability. In contrast, pork from entire males was characterised by significantly higher shear force and shear energy, indicating lower tenderness and reduced culinary value. The longissimus dorsi muscle of entire males contained significantly lower dry matter than that of gilts and barrows and significantly lower crude protein than that of barrows. Meat from entire males was also darker and less yellow than meat from the other sex groups, whereas ultimate pH, ash content, and intramuscular fat content were not significantly affected by sex. Sex significantly influenced the fatty acid profile. Barrows exhibited the highest proportion of unsaturated fatty acids (UFAs), monounsaturated fatty acids (MUFAs), and desirable fatty acids (DFAs), together with the lowest proportion of hypercholesterolemic fatty acids (OFAs), indicating the most favourable nutritional lipid profile. In contrast, entire males were characterised by higher proportions of polyunsaturated fatty acids (PUFAs), including n-6 PUFAs, whereas gilts and barrows contained lower PUFA concentrations. Significant differences were also observed in the concentrations of fat-soluble vitamins. Gilts had higher concentrations of α-, β-, and γ-tocopherols as well as the highest total tocopherol content, suggesting greater antioxidant potential of their meat. Conversely, the longissimus dorsi muscle of entire males contained significantly higher retinol and cholesterol concentrations than the other sex groups. Overall, the results indicate that gilts and barrows produced pork with more desirable technological, sensory, and nutritional characteristics than entire males under the production conditions applied in this study. These findings may contribute to the optimisation of pork production systems and provide useful information for producers and the meat industry when selecting production strategies for DanBred hybrid pigs.

## Figures and Tables

**Table 1:** Ingredient composition of the experimental diets (% as-fed basis).

Ingredient (%)	Finisher 1	Finisher 2	Finisher 3	Finisher 4
Wheat	45.0	45.0	36.0	31.0
Barley	10.0	6.0	5.0	5.0
Maize	0.0	0.0	5.0	5.0
Triticale	18.0	17.0	25.0	20.0
Rye	0.0	10.0	12.0	25.0
Rapeseed meal	8.0	6.5	5.0	2.0
Soybean meal (46% CP)	5.0	1.5	1.5	0.0
Sunflower meal	4.5	4.5	4.5	4.5
Poultry fat	2.4	2.0	1.4	1.4
Limestone	+	+	+	+
Animal protein hydrolysate	+	+	+	+
Crystalline amino acids	+	+	+	+
Vitamin–mineral premix	+	+	+	+
Xylanase and Phytase	+	+	+	+

Abbreviations: CP, crude protein; (+) indicates inclusion of the ingredient in the diet formulation.

**Table 2:** Analyzed chemical composition and calculated net energy of the experimental diets (as-fed basis).

Nutrient	Unit	Finisher 1	Finisher 2	Finisher 3	Finisher 4
Net energy (calculated)	MJ/kg	10.35	10.25	10.10	9.95
Crude protein	g/kg	170.4	154.0	141.3	142.0
Crude fibre	g/kg	39.0	34.0	33.6	38.0
Crude fat	g/kg	44.0	36.0	31.0	28.0
Crude ash	g/kg	47.9	42.0	37.0	36.0
Lysine	g/kg	11.50	10.20	9.12	8.70
Methionine	g/kg	3.80	3.40	3.00	2.90
Calcium	g/kg	6.90	6.00	5.40	4.80
Total phosphorus	g/kg	4.76	4.10	3.80	3.84
Sodium	g/kg	2.00	2.00	1.70	1.70

Data are expressed on an as-fed basis. Net energy values were calculated from diet composition according to the recommendations of the SEGES Pig Research Centre.

**Table 3:** Slaughter performance and carcass characteristics of DanBred hybrid pigs according to sex (mean ± SD).

Trait	Unit	Gilts (*n* = 10)	Barrows (*n* = 10)	Entire Males (*n* = 10)
Final body weight	kg	131.3 ± 9.7	130.7 ± 15.6	127.0 ± 10.6
Carcass weight	kg	104.1 ^a^ ± 8.7	103.3 ^a^ ± 13.0	100.4 ^b^ ± 9.1
Lean meat percentage	%	60.08 ± 2.20	59.58 ± 2.94	59.73 ± 1.98
Backfat thickness (C7)	mm	14.5 ± 3.6	14.2 ± 4.7	12.6 ± 2.0
Loin eye area	mm	69.4 ^A^ ± 7.4	64.5 ^a^ ± 7.4	56.7 ^bB^ ± 8.2

Means within the same row bearing different superscript lowercase letters (a, b) differ significantly at *p* < 0.05. Means within the same row bearing different superscript uppercase letters (A, B) differ significantly at *p* < 0.01.

**Table 4:** Physicochemical characteristics and proximate composition of the longissimus dorsi muscle according to sex (mean ± SD).

Trait	Unit	Gilts (*n* = 10)	Barrows (*n* = 10)	Entire Males (*n* = 10)
Ultimate pH (pH24)	–	5.30 ± 0.11	5.26 ± 0.09	5.29 ± 0.12
Lightness (L*)	–	60.33 ± 1.66	60.89 ^a^ ± 2.18	58.81 ^b^ ± 2.52
Redness (a*)	–	3.37 ± 0.73	3.49 ± 1.11	2.98 ± 0.82
Yellowness (b*)	–	12.88 ^A^ ± 0.41	12.76 ^A^ ± 0.40	12.20 ^B^ ± 0.49
Dry matter	%	25.63 ^a^ ± 0.65	25.75 ^A^ ± 0.58	24.89 ^bB^ ± 0.64
Crude protein	%	22.86 ± 0.40	22.95 ^a^ ± 0.49	22.52 ^b^ ± 0.47
Ash	%	1.17 ± 0.03	1.16 ± 0.03	1.18 ± 0.03
Intramuscular fat	%	1.81 ± 0.67	1.73 ± 0.62	1.27 ± 0.47

Means within a row with different superscript lowercase letters (a, b) differ significantly at *p* < 0.05. Means within a row with different superscript uppercase letters (A, B) differ significantly at *p* < 0.01.

**Table 5:** Fatty acid composition of intramuscular fat in the longissimus dorsi muscle of DanBred hybrid pigs according to sex (g/100 g total fatty acids; mean ± SD).

Fatty Acid	Gilts (*n* = 10)	Barrows (*n* = 10)	Entire Males (*n* = 10)
Lauric acid (C12:0)	0.050 ± 0.008	0.050 ± 0.008	0.051 ± 0.009
Myristic acid (C14:0)	1.048 ± 0.110	1.011 ± 0.093	1.037 ± 0.101
Pentadecanoic acid (C15:0)	0.017 ^b^ ± 0.007	0.017 ^b^ ± 0.005	0.022 ^a^ ± 0.005
Palmitic acid (C16:0)	28.84 ± 1.95	27.19 ± 2.02	28.53 ± 1.90
Palmitoleic acid (C16:1n-7)	3.710 ^a^ ± 0.634	3.549 ± 0.333	3.127 ^b^ ± 0.491
Margaric acid (C17:0)	0.088 ^B^ ± 0.037	0.086 ^B^ ± 0.024	0.131 ^A^ ± 0.024
Heptadecenoic acid (C17:1)	0.092 ^b^ ± 0.026	0.081 ^B^ ± 0.016	0.110 ^Aa^ ± 0.016
Stearic acid (C18:0)	13.97 ± 1.04	13.06 ^B^ ± 0.82	14.82 ^A^ ± 1.37
Oleic acid (C18:1n-9)	42.97 ^ad^ ± 2.04	45.85 ^Ac^ ± 2.43	40.41 ^Bb^ ± 2.51
Linoleic acid (C18:2n-6)	7.210 ^b^ ± 2.206	6.968 ^B^ ± 1.735	9.621 ^Aa^ ± 2.592
α-Linolenic acid (C18:3n-3)	0.211 ^b^ ± 0.054	0.190 ^B^ ± 0.079	0.287 ^Aa^ ± 0.078
Arachidic acid (C20:0)	0.153 ^A^ ± 0.016	0.151 ^A^ ± 0.020	0.129 ^B^ ± 0.010
Gondoic acid (C20:1n-9)	0.539 ± 0.066	0.543 ± 0.093	0.482 ± 0.043
Eicosadienoic acid (C20:2)	0.220 ± 0.067	0.217 ± 0.050	0.273 ± 0.072
Arachidonic acid (C20:4n-6)	0.857 ± 0.404	1.022 ± 0.377	0.941 ± 0.320
Behenic acid (C22:0)	0.019 ± 0.015	0.014 ± 0.011	0.015 ± 0.012
Saturated fatty acids (SFAs)	44.19 ^a^ ± 2.56	41.58 ^b^ ± 2.67	44.75 ^a^ ± 2.88
Unsaturated fatty acids (UFAs)	55.81 ^b^ ± 2.56	58.42 ^a^ ± 2.67	55.25 ^b^ ± 2.88
Monounsaturated fatty acids (MUFAs)	47.31 ^ca^ ± 2.14	50.02 ^Ab^ ± 2.48	44.13 ^Bd^ ± 2.67
Polyunsaturated fatty acids (PUFAs)	8.498 ^b^ ± 2.703	8.398 ^b^ ± 2.071	11.12 ^a^ ± 3.030
n-3 PUFAs	0.211 ^b^ ± 0.054	0.190 ^B^ ± 0.079	0.287 ^Aa^ ± 0.078
n-6 PUFAs	8.287 ^b^ ± 2.650	8.208 ^b^ ± 2.006	10.83 ^a^ ± 2.959
Desirable fatty acids (DFAs = UFAs + C18:0)	69.78 ^b^ ± 1.96	71.48 ^a^ ± 2.08	70.08 ± 1.94
Hypercholesterolemic fatty acids (OFAs = C14:0 + C16:0)	30.22 ^a^ ± 1.96	28.52 ^b^ ± 2.08	29.92 ± 1.94

Means within a row with different superscript lowercase letters (a, b, c, d) differ significantly at *p* < 0.05. Means within a row with different superscript uppercase letters (A, B) differ significantly at *p* < 0.01.

**Table 6:** Concentrations of vitamin A, vitamin E homologues, and cholesterol in the longissimus dorsi muscle of DanBred hybrid pigs according to sex (mean ± SD).

Variable	Unit	Gilts (*n* = 10)	Barrows (*n* = 10)	Entire Males (*n* = 10)
α-Tocopherol	mg/kg	2.026 ^a^ ± 0.652	1.510 ^b^ ± 0.313	1.646 ± 0.389
β-Tocopherol	mg/kg	0.027 ^a^ ± 0.013	0.022 ± 0.005	0.016 ^b^ ± 0.006
γ-Tocopherol	mg/kg	0.136 ^a^ ± 0.036	0.096 ^b^ ± 0.034	0.093 ^b^ ± 0.040
δ-Tocopherol	mg/kg	0.084 ± 0.045	0.053 ± 0.023	0.064 ± 0.034
Total tocopherols	mg/kg	2.273 ^a^ ± 0.708	1.681 ^b^ ± 0.345	1.816 ± 0.416
Retinol (vitamin A)	mg/kg	0.226 ± 0.065	0.195 ^b^ ± 0.045	0.248 ^a^ ± 0.053
Cholesterol	mg/100 g	28.46 ^B^ ± 7.49	31.19 ^B^ ± 4.89	42.97 ^A^ ± 12.31

Means within a row with different superscript lowercase letters (a, b) differ significantly at *p* < 0.05. Means within a row with different superscript uppercase letters (A, B) differ significantly at *p* < 0.01.

**Table 7:** Sensory characteristics of the longissimus dorsi muscle of DanBred hybrid pigs according to sex (mean ± SD).

Sensory Attribute	Unit	Gilts (*n* = 10)	Barrows (*n* = 10)	Entire Males (*n* = 10)
Odor intensity	points	3.90 ± 0.74	3.95 ± 0.96	4.40 ± 0.70
Odor acceptability	points	4.45 ^a^ ± 0.60	4.50 ^a^ ± 0.71	3.60 ^b^ ± 1.15
Tenderness	points	3.30 ± 1.09	3.05 ± 0.86	2.65 ± 1.29
Juiciness	points	3.70 ^a^ ± 0.59	3.15 ^b^ ± 0.24	3.85 ^a^ ± 0.78
Flavor intensity	points	3.80 ^B^ ± 0.79	4.30 ± 0.48	4.60 ^A^ ± 0.52
Flavor acceptability	points	4.35 ^a^ ± 0.58	4.70 ^A^ ± 0.48	3.35 ^bB^ ± 1.42

Means within a row with different superscript lowercase letters (a, b) differ significantly at *p* < 0.05. Means within a row with different superscript uppercase letters (A, B) differ significantly at *p* < 0.01.

**Table 8:** Warner–Bratzler shear force (WBSF) and texture profile analysis (TPA) of the longissimus dorsi muscle according to sex (mean ± SD).

Texture Parameter	Unit	Gilts (*n* = 10)	Barrows (*n* = 10)	Entire Males (*n* = 10)
Warner–Bratzler shear force	N	60.99 ^B^ ± 8.96	58.57 ^B^ ± 8.12	82.48 ^A^ ± 15.18
Shear energy	N/s	166.68 ^B^ ± 19.94	157.93 ^B^ ± 15.07	217.45 ^A^ ± 34.05
Hardness	N	7.884 ± 2.064	7.757 ± 1.064	8.279 ± 1.349
Springiness	–	0.772 ^a^ ± 0.014	0.747 ^b^ ± 0.014	0.741 ^b^ ± 0.021
Cohesiveness	–	0.712 ^a^ ± 0.019	0.687 ^b^ ± 0.019	0.692 ^b^ ± 0.024
Chewiness	N	4.424 ± 0.531	4.015 ± 0.531	4.349 ± 0.837
Resilience	–	0.326 ^a^ ± 0.014	0.309 ^b^ ± 0.014	0.316 ± 0.017

Means within a row with different superscript lowercase letters (a, b) differ significantly at *p* < 0.05. Means within a row with different superscript uppercase letters (A, B) differ significantly at *p* < 0.01.

## Data Availability

The data that support the findings of this study are available on request from the corresponding author (K.K.).
